# Comparing behavioural sampling methods in shelter dogs: implications for enrichment studies

**DOI:** 10.1007/s10071-026-02075-z

**Published:** 2026-06-03

**Authors:** Elena Buso, Maria Loconsole, Simona Normando

**Affiliations:** 1 Department of Comparative Biomedicine and Food Science, University of Padua, Viale dell’Università 16, Agripolis, Legnaro, 35020 PD Italy; 2 Department of General Psychology, University of Padua, Via Venezia, 8, Padova, 35131 PD Italy

**Keywords:** Shelter dogs, *Canis lupus familiaris*, Environmental enrichment, Behavioural recording methods

## Abstract

Animals living in human-dominated environments often face constrains on expression of natural behavioural repertoire and compromised welfare. Domestic dogs (*Canis lupus familiaris*) housed in shelters provide a valuable model for examining both welfare outcomes and methodological approaches to behavioural assessment in an artificial, and human organised, setting. Here we evaluate behavioural sampling methods within an ecologically valid experimental framework. Specifically, we compared continuous recording (CR) and instantaneous sampling (IS; 15s, 30s, and 6s intervals) following enrichment manipulation, reflecting a real-world research context for behavioural data collection. From a methodological perspective, results showed that IS systematically underestimated behavioural expression compared to CR, with increasing underestimation from IS-15 to IS-60. Regarding enrichment, using CR as the most reliable approach, we investigated the short-term behavioural effects of social interaction with conspecifics and food-based olfactory stimulation. We found that, while overall behavioural repertoires did not differ markedly between enrichment conditions, inactive lying increased following social interaction compared to olfactory enrichment. Stress-related behaviours were rare, indicating that neither enrichment type was aversive and that both social and olfactory enrichment can be safely implemented in shelters. Our findings highlight the importance of aligning sampling methods with specific research aims and behavioural outcomes and inform the implementation of different enrichment programs in shelter dogs. This integrated approach aligns with the principle of reduction by maximising information from a single dataset, allowing methodological and experimental conclusions under realistic conditions.

**Supplementary Information:**

The online version contains supplementary material available at 10.1007/s10071-026-02075-z.

## Introduction

Animals in human-dominated environments face rapid and often unpredictable changes, with humans affecting access to resources and space, or expression of natural behaviour and social interaction. Domestic dogs (*Canis lupus familiaris*) living in animal shelters provide a compelling model for studying animal behaviour in a human-created environment. Shelter dogs may experience abrupt environmental change upon relinquishment including loss of control, separation from previous social partners, and confinement in a highly structured setting where humans control access to resources, social interactions, and daily routines. These conditions can negatively affect welfare both acutely and over the long term (Raudies et al. 2021), limiting dogs’ ability to express species-specific behaviours such as exploration and social interactions, which are crucial for their mental and emotional well-being (Stephen and Ledger 2005). Confinement can also trigger stress-related behaviours, ranging from acute indicators such as panting, vocalization, and lip licking (Beerda et al. 1997) to chronic manifestations, including heightened vigilance, yawning, and repetitive behaviours (stereotypies), such as pacing and excessive self-grooming (Hubrecht et al. 1992; Beerda et al. 1998, 1999; Hiby et al. 2006; Raudies et al. 2021). Stress responses in shelter dogs have a high degree of individual variability, since temperament and past experiences highly influence how dogs cope with stressors, including confinement (Beerda et al. 1997). Some dogs may show clear signs of distress, while others may exhibit a lack of positive behaviours, which has also been suggested as an indicator of reduced welfare (Broom and Johnson 2000).

One strategy to improve the welfare of animals in captivity, including shelter dogs, is the implementation of effective forms of enrichment, consisting of adding new environmental conditions and temporary stimuli that give opportunities for species-specific behaviours and fulfil animals’ physiological and social requirement, promoting psychological and physiological well-being (Hunt et al. 2022). Enrichment has been shown to reduce stress, improve behavioural diversity, and positive social interactions (Markowitz 1982; Jensen and Toates 1993; Newberry 1995), and enhance cognitive function (Rosenzweig and Bennett 1996; van Praag et al. 2000; Wolfer et al. 2004) across multiple species and settings. These include for example lab rats, *Rattus norvegicus* (Simpson and Kelly 2011) and mice, *Mus musculus* (Wolfer et al. 2004); farm pigs, *Sus scrofa* (Godyń et al. 2019), broiler chickens, *Gallus gallus* (Vas et al. 2023), and laying hens (Xu et al. 2022); house cats, *Felis catus* (Herron and Buffington 2010); and captive geckos, *Eublepharis macularius* (Zieliński 2023). In dogs, enrichment programs (ranging from inanimate objects such as toys and sensory stimuli, to animated forms including interactions with humans and conspecifics) have been linked to increased relaxation, reduced undesirable behaviours, and improved welfare outcomes (Wells 2004a; Herron et al. 2014; Hunt et al. 2022). Dogs exposed to multi-faceted enrichment regimes display more relaxed body postures, are quieter when approached by people, and show less jumping activity; all these traits are associated with decreased stress levels and improved welfare states (Coppola et al. 2006; Herron et al. 2014).

An essential step in implementing good environmental enrichment is a rigorous and efficient monitoring of its effects (Bloomsmith et al. 1991). The choice of data collection methods is crucial in ethological research as it can critically influence the accuracy of behavioural observations (Altmann 1974; Jauhiainen and Korhonen 2005; Ledgerwood et al. 2010; Quirke and O’Riordan 2013; Ceballos et al. 2021; Bateson and Martin 2021; Brereton et al. 2021). One important issue is the choice of whether recording behaviour continuously (continuous recording; CR) or at specific time intervals (instantaneous sampling IS). CR provides detailed data but is time-intensive, while IS at fixed intervals risks missing behaviours and increasing variability (Daigle and Siegford 2014; Chen et al. 2019; Bateson and Martin 2021; Brereton et al. 2021). Understanding how different sampling approaches affect behavioural data is therefore essential for reliably assessing interventions.

In the present study, we addressed two distinct research questions. First, we tackled a methodological question: whether IS at intervals of 15 s, 30 s, and 60 s captures the range of behaviours expressed by shelter dogs as effectively as CR. This comparison provides a direct evaluation of how different sampling approaches influence behavioural data collection and has the potential to inform best practices for future studies of dog behaviour. This methodological aim is embedded within an ecologically valid experimental framework, reflecting realistic research circumstances in which behavioural data are typically collected. Second, using CR, we explored an applied question: the short-term behavioural effects of two types of environmental enrichment, social interactions with conspecifics and food-based olfactory stimulation, on shelter dogs. Together, these questions allow us to both contribute to methodological knowledge on behaviour sampling while also assessing how different enrichment strategies affect welfare-relevant behaviours in a human-controlled environment.

## Materials and methods

### Subjects and housing at the shelter

The study was conducted at the *Parco Zoofilo San Francesco*, a dog shelter located in Presina di Piazzola sul Brenta (PD), Italy. This facility accommodates stray and relinquished dogs from 28 municipalities within the former *Ulss n.15 Alta Padovana*, serving a population of over 250,000 residents. The shelter comprises 59 enclosures, classified according to function: 12 sanitary enclosures for newly admitted dogs or those requiring medical isolation, 29 permanent enclosures for long-term housing, and 18 modified enclosures, which have the same structure as the permanent enclosures but are divided in half, creating two smaller sections.

Dogs were housed either individually or in groups, depending on their health, and behavioural needs, and social compatibility. Dogs participating in the study were individually housed in three types of pens. Whole pens had a total area of 53.54 square metres and included both indoor and outdoor spaces. The indoor area measured 8.06 square metres, was concrete paved, and contained a wooden double doghouse and an automated water dispenser. The outdoor area included an uncovered section of 37.87 square metres, with a lateral concrete paved area and a central pebble paved area, as well as a 7.61 square metre concrete paved section covered by a canopy, which also contained a wooden double doghouse. Half exterior pens had a total area of 23.89 square metres and consisted of an uncovered area of 16.28 square metres, pebble paved with a concrete paved corridor, and a 7.61 square metre concrete paved area covered by a canopy, equipped with a wooden double doghouse and an automated water dispenser. Half interior pens had a total area of 29.66 square metres and included both indoor and outdoor spaces. The indoor area measured 8.06 square metres, was concrete paved, and contained a wooden double doghouse and an automated water dispenser. The outdoor area consisted of an uncovered 21.6 square metre space, with a lateral concrete paved section and a central pebble paved area.

Wire mesh fences separating the pens allowed visual contact between dogs. The facility also includes an outdoor exercise area divided into four fenced zones, where dogs are allowed supervised access for approximately one hour per week. Routine activities at the shelter include daily cleaning of enclosures in the morning, feeding once per day around 11:00 AM, free access to water, and regular veterinary care.

During the study period, the shelter housed an average of 90 dogs. Of these, 16 animals were included in the study (Table 1). Dogs were selected based on the following inclusion criteria: being housed individually in permanent enclosures, being in good general health, having no medical conditions likely to affect natural behaviour, and having no prior history of severe behavioural problems such as aggression or food-related resource guarding. Dogs that did not meet all of these criteria were excluded from the study.

**Table 1:** Individual and housing specifications of the dogs included in the study

Name	Approximate age (in years)	Sex	Breed	Permanence (in months)	Housing (pen)
Bianca	5	F	Maremma Shepherd	16	Whole
Tommy	8	M	Crossbred	16	Half exterior
Ray	7	M	Rottweiler	14	Half exterior
Masha	9	F	Cane Corso	55	Whole
Beeper	6	M	Setter	2	Half exterior
Filo	11	M	Crossbred	34	Half exterior
Freddy	7	M	Crossbred	15	Whole
Sole (1)	9	M	Maremma Shepherd	35	Half interior
Jimmy	9	M	Crossbred	20	Half interior
Raul	2	M	Border collie mix	17	Whole
Tyson	5	M	Amstaff mix	35	Whole
Gaston	6	M	Amstaff	26	Whole
Loreggian	8	M	Crossbred	92	Half exterior
Sole (2)	8	M	Maremma Shepherd	32	Whole
George	3	M	Dogo Amstaff mix	29	Whole
Ringhio	11	M	Cocker mix	105	Whole

### Environmental enrichment

All dogs participated in two different environmental enrichment sessions, once per enrichment. Each individual experienced both enrichments, with a minimum interval of five and a maximum of nine days between sessions. Dogs were randomly assigned to one of two groups to counterbalance the order in which they received each enrichment type. Enrichment sessions were conducted inside the dogs’ home enclosures in the afternoon, between 3pm and 7pm. Sessions were rescheduled in cases of adverse weather conditions, extreme heat, or logistical constraints (e.g., veterinary procedures or other events likely to affect behaviour). Each dog experienced five minutes of the enrichment, followed by a separate period of observation.

For the human social interaction enrichment, an operator (either shelter staff or volunteer at the shelter) entered the enclosure and interacted directly with the dog for the entire session, allowing the first minute for the dog to gradually acclimatise to their presence. Interaction consisted of petting when accepted by the dog or, when physical contact was not sought, calm companionship such as sitting and speaking softly to the dog.

For the olfactory search enrichment, the operator first entered the enclosure and allowed the dog one minute to acclimatise to their presence. The dog was then presented with a scent-based foraging task lasting four minutes. The task consisted of a large bowl (approximately 25 cm in diameter and 15 cm in height) filled with crumpled fabric items (e.g. small towels or pillowcases) in which food rewards (pieces of sausage) were hidden. If the dog completed the search before the end of the four-minute period, the operator replenished the bowl with additional food rewards to maintain engagement.

### Behavioural observations recording

Behavioural recordings were collected between May and August 2023, across a total of 18 observation days, with a maximum of three videos recorded per day. All observations were conducted using video recordings obtained with a Samsung Galaxy S10e smartphone. The device was secured outside the enclosure using a metal wire support and positioned to maximise visibility while minimising blind spots, adapting to the structure of each enclosure. The camera was set to 0.5× zoom to maximise the recorded area.

Each recording session began immediately after the operator exited the enclosure following the enrichment activity, to capture short-term post-enrichment behavioural responses. To minimise external disturbances, shelter staff were asked to avoid walking near the enclosure during recording. The first 20 s of footage were excluded to avoid capturing any behavioural changes associated with the operator’s departure, and analyses were conducted on the subsequent 10 min of recording.

### Behavioural scoring and ethogram

Behavioural observations were based on a working ethogram that was specifically developed for the purpose of this study. The ethogram was informed by existing literature on behavioural analysis in shelter dogs under conditions of chronic confinement (Schipper et al. 2008; Normando et al. 2014; Protopopova et al. 2014, 2018; Owczarczak-Garstecka and Burman 2016; Walker et al. 2016; Murtagh et al. 2020; Raudies et al. 2021). These sources guided the identification of relevant behavioural categories, which were further refined through direct observation of the study subjects in the shelter environment. The final working ethogram (**Supplementary table T1**) comprised 47 behaviours. Statistical analyses were restricted to behaviours occurring in at least 50% of the videos to reduce zero inflation and ensure adequate representation.

The scoring accuracy for continuous recording was set at the 1 s level, meaning that only completed seconds were recorded (i.e., the counter on the video only changed when a complete second had elapsed). Instantaneous sampling data were extracted from continuous recordings by coding the behaviour observed at predefined sampling points. This approach was chosen over conducting separate instantaneous observations to minimize observer bias, particularly for behaviours that were easily detectable and occurred immediately before or after the sampling point.

### Intra-observer reliability test

To assess the reliability of behavioural observations, an intra-observer reliability test was conducted. A total of 43 video segments, each lasting 2 min, were randomly selected and re-analysed by the same observer blind to the experimental manipulation. Reliability was assessed for behaviours scored using the focal continuous recording method by comparing the first and second coding of each video segment.

Intra-observer reliability was quantified using the Intraclass Correlation Coefficient (ICC), with values greater than 0.9 considered indicative of high agreement. When the full dataset was analysed, including zero values corresponding to behaviours not observed, the ICC was 1 (F_5589, 5590_ = 495,755, *p* < 0.001, 95% CI: 1–1). An additional analysis was conducted excluding zero values to control for possible inflation of agreement estimates. This second analysis also yielded an ICC of 1 (F_924, 925_ = 352,729, *p* < 0.0001, 95% CI: 1–1), indicating a very high level of consistency in behavioural scoring.

## Dara analysis and results

Statistical analysis were run using R 4.4.0 (R Core Team 2022). All graphs were generated using the ggplot2 package (Wickham 2010). The dataset generated during this study is available as **Supplementary material S1.**

### Comparison between different recording methods: data analysis

**Fig. 1:**
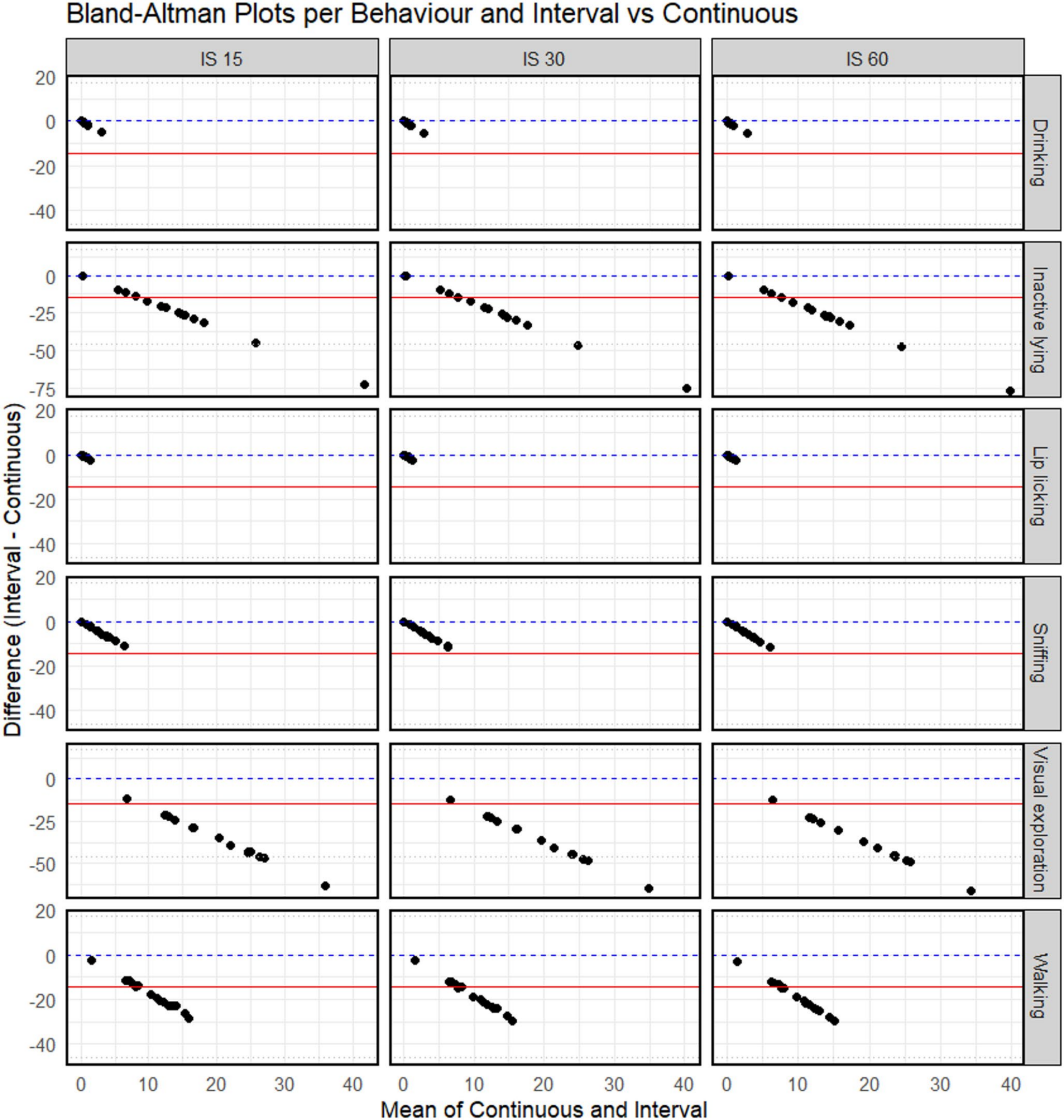
Bland–Altman plots comparing interval sampling methods (SI 15, SI 30, SI 60) with continuous recording for each behaviour. Panels are arranged by behaviour (rows) and sampling interval (columns). For each dog, points represent the difference between interval sampling and continuous recording (Interval − Continuous) plotted against their mean value. The blue dashed line indicates zero difference. Red solid lines show the mean bias for each behaviour and interval. Negative values indicate a systematic over underestimation by IS relative to CR

First, we wanted to explore whether continuous, 15- 30- and 60- seconds IS methods differ in their ability of describing different behaviours in dogs. Agreement between CR and each IS method was assessed using Bland–Altman plots (Fig. 1) and linear mixed effects model. Behavioural measures were expressed as the percentage of the 10-min observation period rather than raw values. This transformation was applied to allow direct comparison across behaviours and sampling methods on a common scale and to facilitate interpretation of differences between methods. Bland–Altman plots were used to visually assess agreement between IS and CR by plotting the difference between methods (IS − continuous) against their mean, in line with recent practices in veterinary science and animal behaviour (Cartoni Mancinelli et al. 2023; Moore 2024).

To formally test whether behavioural estimates differed as a function of sampling method and behaviour, we fitted a linear mixed-effects model (R package: lme4 (Bates et al. 2015) with behavioural value as the response variable. Sampling method (CR, IS 15, IS 30, IS 60), behaviour, and their interaction were included as fixed effects, and subject identity was included as a random intercept to account for repeated measures. CR was used as the reference level. Model assumptions were evaluated using simulation-based residual diagnostics (R package: DHARMa (Hartig and Lohse 2022). Log-transformation of the response variable improved residual distributions and reduced heteroscedasticity. While minor deviations from the expected distribution remained, these were not considered severe. Type III ANOVA was used to assess the significance of fixed effects. Post-hoc estimated marginal means (R package: emmeans (Lenth 2022) were computed for each IS within each behaviour, and Dunnett-adjusted contrasts were used to compare each IS method directly against CR.

### Comparison between different recording methods: results

The mixed-effects model revealed significant main effects of behaviour (F₅,₇₄₄ = 205.46, *p* < 0.001) and sampling method (F₃,₇₄₄ = 397.437, *p* < 0.001), as well as a significant behaviour × sampling method interaction (F₁₅,₇₄₄ = 11.11, *p* < 0.001), indicating that the impact of sampling method differed across behaviours.

Post-hoc EMM contrasts (R package: emmeans (Lenth 2022) confirmed that IS led to systematic underestimation of behavioural durations relative to continuous recording (Table 2). All IS methods yielded significantly lower estimates than CR (all *p* < 0.001), with a gradient of increasing underestimation from IS15 to IS60.

**Table 2:** Post-hoc contrasts comparing IS to CR for each behaviour. Estimates are derived from a linear mixed-effects model fitted on log-transformed data and represent differences between IS methods and CR. Percent underestimation values are calculated from back-transformed estimates and indicate the proportional decrease in recorded behaviour duration relative to CR

Contrast	Behaviour	estimate	t ratio	% Underestimation
IS15 – Cont	Drinking	-1.229	-6.16	70.7
IS30 – Cont		-1.393	-6.99	75.2
IS60 – Cont		-1.393	-6.99	75.2
IS15 – Cont	Inactive lying	-2.072	-10.39	87.4
IS30 – Cont		-2.524	-12.66	92.0
IS60 – Cont		-2.895	-14.52	94.5
IS15 – Cont	Lip licking	-0.997	-5.00	63.1
IS30 – Cont		-1.075	-5.39	65.9
IS60 – Cont		-1.140	-5.72	68.0
IS15 – Cont	Sniffing	-2.046	-10.26	87.1
IS30 – Cont		-2.307	-11.57	90.0
IS60 – Cont		-2.559	-12.84	92.3
IS15 – Cont	Visual exploration	-2.600	-13.04	92.6
IS30 – Cont		-3.215	-16.12	96.0
IS60 – Cont		-3.838	-19.25	97.8
IS15 – Cont	Walking	-2.457	-12.32	91.4
IS30 – Cont		-2.987	-14.98	95.0
IS60 – Cont		-3.463	-17.37	96.9

### Effect of different enrichments on dogs’ behaviour

To assess whether environmental enrichment affected behaviour, analyses were conducted using CR data only, as this method provides the most complete and unbiased behavioural estimates.

Behavioural values were analysed using a linear mixed-effects model, with behaviour, enrichment condition, and their interaction included as fixed effects. Subject identity was included as a random intercept to account for repeated measurements across behaviours within individuals. The response variable was log-transformed to improve normality and homoscedasticity of residuals, as confirmed by simulation-based diagnostics (R package: DHARMa (Hartig and Lohse 2022). The mixed-effects model revealed a strong main effect of behaviour (F₅,₁₈₀ = 57.07, *p* < 0.001), reflecting substantial differences in the overall expression of behaviours. In contrast, there was no significant main effect of enrichment (F₁,₁₈₀ = 0.01, *p* = 0.97), indicating that enrichment did not produce a uniform change in behaviour across the repertoire. The Behaviour × Enrichment interaction is to be considered conventionally non-significant (F₅,₁₈₀ = 2.21, *p* = 0.05).

**Fig. 2:**
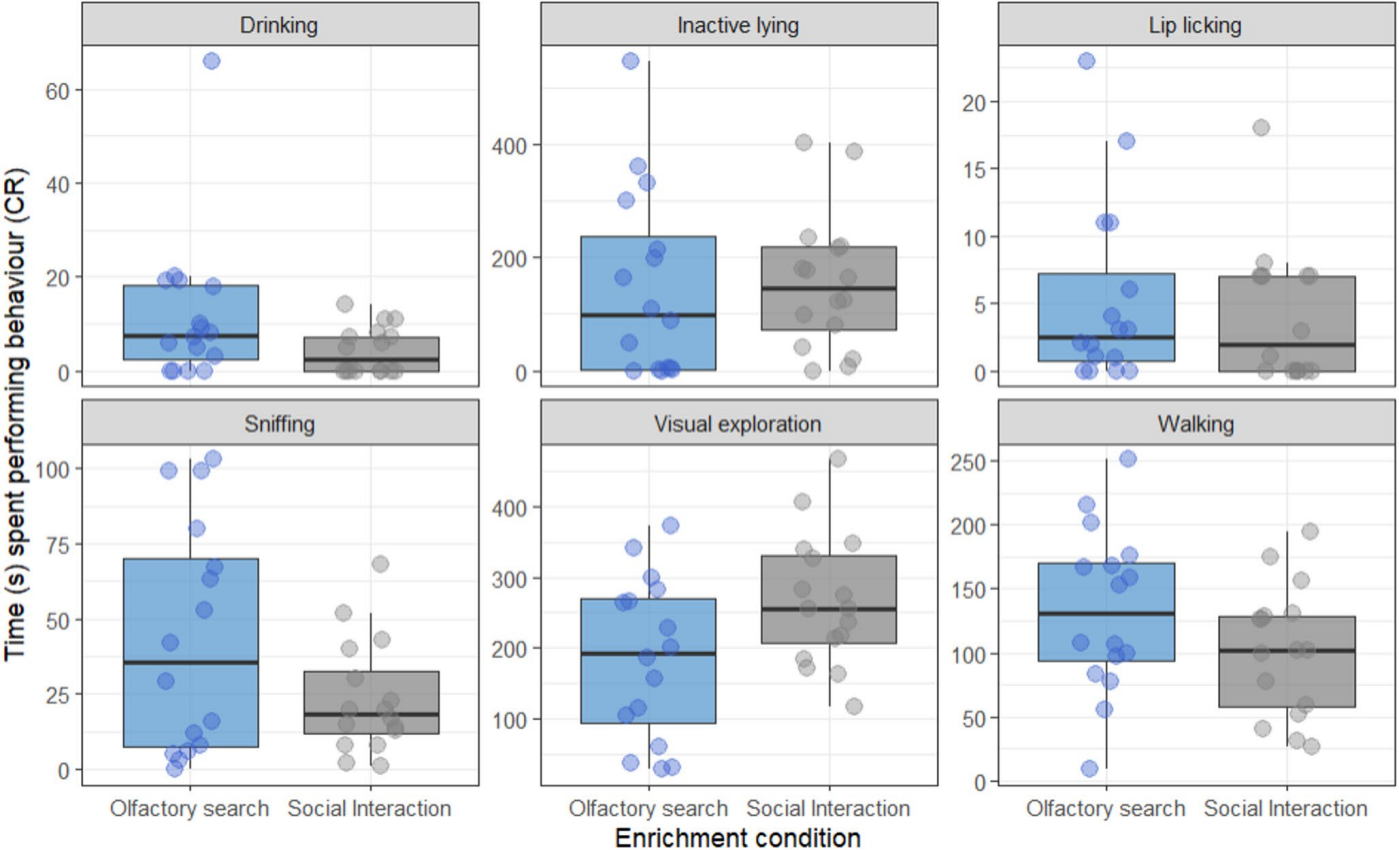
Time spent for each behaviour obtained from continuous recording under the two enrichment conditions. Boxplots show the median and interquartile range for each behaviour, with individual observations overlaid as jittered points. Panels correspond to the six behavioural categories. Colours indicate enrichment condition: in grey social interaction, and in blue olfactory search

Pairwise contrasts (R package: emmeans (Lenth 2022) based on the log transformed model revealed limited evidence for differences between enrichment conditions across behaviours (Fig. 2). For drinking, the contrast between olfactory search and social interaction was not significant (estimate = 0.78, SE = 0.44, t = 1.79, *p* = 0.08), although a tendency towards higher levels following olfactory enrichment can be observed. Inactive lying showed a statistically significant difference (estimate = -0.99, t = -2.260, *p* = 0.025), indicating lower levels following olfactory search compared to social interaction. No significant differences were found for lip licking (estimate = 0.21, t = 0.48, *p* = 0.63), sniffing (estimate = 0.31, t = 0.7, *p* = 0.49), visual exploration (estimate = -0.57, t = -1.31, *p* = 0.19), or walking (estimate = 0.22, t = 0.52, *p* = 0.6). We recommend interpreting these results with caution as the overall effect of enrichment condition was not statistically significant at the model level. As such, the isolated significant contrast for inactive lying may reflect a Type I error, and the general pattern is more consistent with weak or limited behavioural differences between enrichment types. Together, these findings suggest that, while some behaviours may show condition specific trends, there is no strong evidence for robust short-term effects of enrichment on the behavioural repertoire under the present conditions.

## Discussion

The present study addressed two distinct but complementary questions: (i) how different behavioural sampling methods influence the estimation of dog behaviour in a shelter context, and (ii) whether two forms of environmental enrichment produce different short-term behavioural effects in shelter dogs when assessed using CR as the most reliable recording approach. Although these questions were conceptually distinct, they were examined within a single ecologically valid experimental framework, allowing methodological evaluation to be embedded within an applied welfare manipulation rather than conducted in isolation. This integrated design strengthens the relevance of the findings for real world behavioural research, where data collection typically occurs in dynamic and applied settings.

By separating these questions analytically while maintaining a unified experimental context, the study contributes both methodological and applied insights relevant to behavioural research and welfare assessment in human-controlled environments. This approach of evaluating sampling methods while also assessing enrichment without compromising rigour reduces the need for parallel studies, maximises the informational yield of a single dataset, and supports a more efficient and ethically aligned research strategy in line with the principle of reduction.

A first focus was on the methodological implications of the recording rule choice. While CR is often considered the gold standard in ethological studies, it can also result time consuming or challenging for the researcher to ensure accuracy, especially where large amounts of data (either in terms of duration or number of individuals) are being recorded (Hämäläinen et al. 2016; Brereton et al. 2022). An alternative to CR is IS, where one or more responses are recorded at preselected moments in time (e.g., every 15 s) (Bateson and Martin 2021; Brereton et al. 2022).

Our results showed that IS systematically underestimate the behaviour compared to CR. Upon inspection of the Bland-Altman graph, it is notable that some behaviours (i.e., inactive lying, visual exploration, and walking) show large negative differences, meaning that IS severely underestimates the behaviour compared to CR. This effect seems less pronounced, although still noticeable for drinking and lip licking, and sniffing. Statistical analysis confirmed this pattern, by showing that IS (at all three intervals) systematically underestimate behavioural durations across all behaviours, with increasing underestimation from IS-15 to IS-60. These results extend previous methodological work by demonstrating that estimation biases can occur even under controlled conditions and when sampling intervals are relatively short. While IS offers clear practical advantages in terms of efficiency, its use requires careful alignment between the research question, the behavioural repertoire of interest, and the sampling interval adopted. For studies aiming to quantify time budgets or sustained behavioural states, CR remains the most reliable approach. Conversely, IS may be appropriate when the focus is on broad behavioural trends (Bateson and Martin 2021) or when logistical constraints preclude continuous observation, provided its limitations are acknowledged.

As a second aim of this study, we also explored the short-term effects of enrichment in a human-controlled environment. Using CR to avoid measurement artefacts, we assessed the short-term behavioural effects of two enrichment strategies in shelter dogs. Overall, behavioural expression was primarily shaped by the nature of the behaviours themselves rather than by enrichment condition, indicating that a single, brief enrichment session does not uniformly alter dogs’ behavioural repertoires. This is consistent with previous research suggesting that enrichment effects are often behaviour-specific and context-dependent, particularly when assessed over short time frames (Herron and Buffington 2010; Herron et al. 2014). Nevertheless, a clear enrichment-specific effect emerged for inactive lying, which increased following social interaction compared to olfactory search enrichment. However, this finding should be interpreted with caution, given that the overall Behaviour × Enrichment interaction did not reach conventional levels of statistical significance and that multiple comparisons were conducted, increasing the risk of Type I error. As such, this result may reflect a spurious effect and requires further confirmation. If robust, this pattern may be due to a combined action of olfactory search enrichment increasing motor arousal, hence prompting further exploration of the environment and reducing time spent inactive, and social interaction heightening engagement and affiliative processes, possibly inducing a calmer and more relaxed state (Hennessy et al. 1998; Coppola et al. 2006).

Importantly, our study was designed to compare the relative impact of two forms of enrichment under controlled conditions, rather than to assess changes from a neutral or resting state. Comparing two active enrichment conditions allowed us to evaluate their relative effects under controlled and comparable circumstances, minimizing variability related to prior experiences, arousal state, or individual differences that can strongly influence behaviour in shelter dogs and are more likely to emerge in neutral circumstances. While this design brings the limitation of not allowing for direct inference about behavioural changes relative to a resting baseline, it also avoids assumptions about what constitutes a neutral state in a shelter environment, where dogs’ behavioural expression may be shaped by immediately preceding events and individual history. Within this framework, the lack of marked behavioural differences in overall behavioural expression indicates that both enrichment types were similarly effective supporting behavioural engagement without evident signs of stress. Indeed, stress-associated behaviours and stereotypies were rare and often insufficiently expressed to be included in the analysis, indicating that neither enrichment condition was aversive. Future studies incorporating baseline measures would help to more precisely quantify the absolute effects of different enrichment strategies on welfare.

Taken together, these results suggest that both social and olfactory enrichment can be implemented safely in shelter settings, supporting behavioural engagement and welfare. Given the potential for social enrichment to enhance visibility and human-directed attention (Hennessy et al. 1998; Coppola et al. 2006), and for olfactory enrichment to provide cognitive stimulation (Wells 2004b; Hunt et al. 2022), incorporating multiple enrichment strategies may be particularly beneficial for promoting positive behavioural states and possibly improving adoption-relevant outcomes .

## Conclusions

Overall, this study provides both methodological and applied insights into the assessment of shelter dog behaviour in human controlled environments. Our findings indicate that IS systematically underestimates behavioural expression compared to CR, highlighting the importance of selecting sampling methods that align with specific research aims. At the same time, the effects of enrichment were limited and variable across behaviours, with only weak and context dependent differences emerging between social and olfactory conditions. Our two research questions emphasise the value of integrating methodological validation within ecologically relevant experimental frameworks, in line with both scientific and ethical considerations.

## Supplementary Information

Below is the link to the electronic supplementary material.

Supplementary Material 1

Supplementary Material 2

## Data Availability

The dataset generated during this study is available as supplementary material S1 .
