# Supplementary material for: Comparing behavioural sampling methods in shelter dogs: implications for enrichment studies
Source: Anim Cogn. 2026 Jun 3;29(1):55. doi: 10.1007/s10071-026-02075-z (PMC13453999; doi:10.1007/s10071-026-02075-z)
Supplement: Supplementary file 1 [file 10071_2026_2075_MOESM1_ESM.docx]

**Supplementary Table 1: working ethogram.** The final working ethogram consisted of 47 non-mutually exclusive behaviours, organised into nine behavioural categories. These categories were further classified into three groups: active behaviours (seven categories), inactive behaviours (one category), and out-of-sight behaviours (one category). If both the behaviour at the beginning and end of the "not visible" out-of-sight period was the same, and the out-of-sight period lasted no more than 2 seconds, those seconds were assigned to the previously occurring behaviour, otherwise they were classified as "not visible" out-of-sight. Two modifiers were included: position within the enclosure (frontal vs. rear section of the enclosure) and panting, which was excluded as an independent behaviour due to its potential misinterpretation under high temperatures recorded during the study period (May to August 2023, with peaks of 38.7°C). Instead, panting was noted as a modifier to avoid incorrect classification as a stress-related behaviour.

| **Category** | **Behaviour** | | **Description** |
| --- | --- | --- | --- |
| **Active behaviours**: locomotor behaviours | Walking | | Forward movement using the legs, causing the whole body to shift to a new position within the enclosure; no more than one paw is lifted off the ground at any given time |
|  | Running | | A faster form of walking, where multiple paws leave the ground simultaneously |
|  | Standing | | All four paws on the ground, supporting the body in an upright position (in absence of other behaviours) |
|  | Sitting | | The hind legs rest on the ground while the two front legs provide support |
|  | Resting position | | Lying down ventrally or laterally with all four legs resting on and in contact with the ground; the dog may also be curled up in a tight ball. The head may rest on the ground or be held up, and the eyes may be open or closed |
|  | Jumping | | The dog lifts itself off the ground with both hind legs while placing both front legs against the wall or fence bars. This behaviour also includes cases where the dog is observed with all four paws lifted off the ground |
|  | Stretching | | The dog moves its body into an arched position by extending the front legs forward |
|  | Rolling on the ground | | The dog lies on its back and performs wavelike movements with the whole body |
|  | Bite/play with blankets | | The dog bites or plays with blankets |
|  | Solitary play | | The dog plays alone or interacts with a toy provided by the shelter staff |
|  | Beg position | | The front paws are lifted off the ground simultaneously while the hind legs remain bent |
|  | Digging in the ground | | The dog repeatedly scratches the ground with its front paws, creating a hole |
|  | Tail wagging | | Repetitive side-to-side movement of the tail |
|  | Dig/play with water | | The dog digs in the water bowl (placing the distal end of one or both forelimbs into the bowl so that they make contact with the water and performing rhythmic anteroposterior movements to create currents in the water) or plays in the water bowl or the small pool present in the enclosure |
| **Active behaviours**: enviromental exploration | Sniffing | | Forced inhalation of air through the nose |
|  | Visual exploration | | The subject observes specific objects inside or outside the enclosure (not other subjects) |
| **Active behaviours**: manteinance behaviours | Eating | | The dog eats food provided in the enclosure by the staff |
|  | Drinking | | The dog drinks from the provided bowl or an automatic water system |
|  | Urinating | | The dog expels urine |
|  | Defecating | | The dog expels fecal matter |
|  | Self-grooming | | Self-directed grooming behaviors, including licking, self-biting, and scratching, aimed at cleaning and maintaining the coat or fur |
| **Active behaviours**: fear and discomfort behaviours | Cowering | | Curling up or crouching due to fear |
|  | Shaking | | Continuous vibrating movements of the body or part of it |
|  | Lip licking | | A part of the tongue is shown and moved along the upper lip |
|  | Body shake | | Rapid vibration of the entire body |
|  | Yawning | | The mouth opens wide for a few seconds while exhaling |
|  | Biting the fence | | The dog bites the enclosure fence |
|  | Vomiting | | Forced expulsion of stomach contents through the mouth |
|  | Coprophagia | | The dog ingests its own feces or those of another dog |
|  | Digging without purpose | | The dog repeatedly scratches walls, fences, or floors with its front paws |
|  | Stereotypic behaviours | Circular movement | A circular motion is repeatedly traced (>3 times) in one direction or in place |
|  |  | Pacing | The subject walks repetitively within the enclosure following the same trajectory without a specific destination |
|  |  | Tail chasing | Circular body movement aimed at biting the dog's own tail |
| **Active behaviours**: vocalizations | Barking | | The mouth opens and closes rapidly in a snapping motion, producing a short, low-frequency vocalization, often repeated in succession |
|  | Howling | | A continuous, medium-pitched vocalization performed with the muzzle pointed upward |
|  | Whining | | A soft, high-pitched, whistling vocalization occurring in brief, repeated bursts |
|  | Yelping | | A high-pitched, loud, and brief vocalization, stronger than whining |
|  | Growling | | A low, threatening vocalization, often accompanied by baring teeth |
| **Active behaviours**: social behaviours | Positive interaction | | Friendly or playful interaction |
|  | Negative interaction | | Threatening interaction with a rigid or defensive posture |
| **Active behaviours**: other | Sneezing | | Deep inhalation followed by a forced and rapid exhalation through the nostrils |
|  | Paw lift | | The front leg is lifted and lowered, often in rapid succession |
| **Inactive behaviours**  (the dog remains stationary in a specific position, except for small movements, and is not engaged in any other active behaviour) | Standing | | All four paws on the ground, supporting the body in an upright position (in absence of other behaviours) |
|  | Sitting | | The hind legs rest on the ground while the two front legs provide support (in absence of other behaviours) |
|  | Resting position | | Lying down ventrally or laterally with all four legs resting on and in contact with the ground; the dog may also be curled up in a tight ball. The head may rest on the ground or be held up, and the eyes can be open or closed (in absence of other behaviours) |
| **Out-of-sight behaviours** | Not visible | | The dog is not visible to the observer as it is hidden behind objects/walls inside the enclosure |
|  | Inside the doghouse | | The dog is not visible to the observer as it is inside a doghouse |
